# Fast Location and Recognition of Green Apple Based on RGB-D Image

**DOI:** 10.3389/fpls.2022.864458

**Published:** 2022-06-09

**Authors:** Meili Sun, Liancheng Xu, Rong Luo, Yuqi Lu, Weikuan Jia

**Affiliations:** ^1^School of Information Science and Engineering, Shandong Normal University, Jinan, China; ^2^State Key Laboratory of Biobased Materials and Green Papermaking, Qilu University of Technology (Shandong Academy of Sciences), Jinan, China; ^3^Key Laboratory of Facility Agriculture Measurement and Control Technology and Equipment of Machinery Industry, Zhenjiang, China

**Keywords:** green target fruit, center location, density peak clustering, kernel density estimation, RGB-D image

## Abstract

In the process of green apple harvesting or yield estimation, affected by the factors, such as fruit color, light, and orchard environment, the accurate recognition and fast location of the target fruit brings tremendous challenges to the vision system. In this article, we improve a density peak cluster segmentation algorithm for RGB images with the help of a gradient field of depth images to locate and recognize target fruit. Specifically, the image depth information is adopted to analyze the gradient field of the target image. The vorticity center and two-dimensional plane projection are constructed to realize the accurate center location. Next, an optimized density peak clustering algorithm is applied to segment the target image, where a kernel density estimation is utilized to optimize the segmentation algorithm, and a double sort algorithm is applied to efficiently obtain the accurate segmentation area of the target image. Finally, the segmentation area with the circle center is the target fruit area, and the maximum value method is employed to determine the radius. The above two results are merged to achieve the contour fitting of the target fruits. The novel method is designed without iteration, classifier, and several samples, which has greatly improved operating efficiency. The experimental results show that the presented method significantly improves accuracy and efficiency. Meanwhile, this new method deserves further promotion.

## Introduction

The machine vision system, employed to realize the localization and recognition of target fruits, has been widely applied in agricultural production processes, such as apple orchard yield estimation (Maheswari et al., 2021), apple automatic harvesting (Jia et al., 2020a), and fruit growth monitoring (Genno and Kobayashi, 2019). How to achieve accurate recognition and fast location of target fruits will directly affect the reliability and real-time performance of automated operations. This is also the key to research, which has attracted the attention of many domestic and foreign scholars. In the recent years, whether it is with the help of the monocular vision system (Zhang et al., 2017) or binocular vision system (Si et al., 2015), a single fruit (Silwal et al., 2017) or overlapping occluded fruit (Lv et al., 2019b), a static target (Häni et al., 2020a), or a dynamic target (He et al., 2017), it has made great progress in recognition, and most of these studies are implemented with red apples as the harvesting target. However, many types of apples are green apples, such as white winter Pearmain and Granny Smith. Because the color of the fruit is similar to the color of the background branches and leaves, it is difficult to recognize and locate the target fruit. Besides, affected by factors, such as complicated unstructured orchard environment and the blurred borders of irregular bright areas, the performance of visual system is relatively not good.

It is very necessary to improve the recognition and location efficiency of green apples and further improve the automated assembly level of the vision system in agricultural production management. Accurate location and fast recognition of green target fruit become a new challenge. With the joint efforts of many scholars, certain progress has been made (Lv et al., 2019a; Sun et al., 2019; Behera et al., 2020; Ji et al., 2020). It is difficult to recognize green apples only from the perspective of color, and it needs to be processed or try other features. Therefore, Zhang used a color camera equipped with a ring flash to capture images of apple trees. By analyzing the feature difference between the green apple areas and the similar-color background, a classifier was designed based on color features to recognize the green apples in the near-color background. The recognition success rate reached 89.3%, and the algorithm took 3.14s on average (Zhang et al., 2014).

To recognize the same color fruits under unstable light and the occlusion of branches and leaves, Barnea proposed a 3D detection model based on RGB color features and related shape features (Barnea et al., 2016), where the mean average precision of recognition reached 55%. Li DH et al. presented an improved spectral clustering algorithm based on mean shift and sparse matrix to recognize overlapping green apples and the algorithm improved the running speed (Li et al., 2019), The high coincidence degree of the optimized spectral clustering algorithm reached 95.41%, and the false detection rate is 3.05%. Gaussian linear fitting on the foreground images of the green apples was adopted under the V channel, and threshold segmentation was used to segment the images (Li et al., 2018), which was 91.84% of the recognition rate. The gPb-OWT-UCM edge detection algorithm was applied in the green apple detection model based on the SUN saliency detection mechanism by Wang, which can obtain complete and accurate green apple segmentation images (Wang et al., 2019). The above method reached accurate segmentation with average sensitivity, false-positive rate, false-negative rate, and overlapped rate of 8.4, 0.8, 7.5, and 90.5%, respectively. The segmentation time of each apple image was around 37.1 s. All these methods merged corresponding texture features through color features. Due to the changes in light intensity and angle, the boundary between the target fruit and the background is blurred, and the recognition effect is not ideal. Affected by factors, such as occlusion of branches and leaves and overlapping between fruits, some features are absent, which makes the recognition difficult. Some algorithms have high time and space complexity, and it is difficult to meet real-time operation requirements.

With the rapid development of deep learning theory, end-to-end automatic detection process based on deep networks and the advantages of deep extraction of image features (Kamilaris and Prenafeta-Boldú, 2018; Koirala et al., 2019; Zhao et al., 2019; Tang et al., 2020) bring a new perspective for recognizing green apples. A multi-scale multilayer perceptron and a convolutional neural network (CNN) were applied to segment apple images and extract the apple target by Bargoti, where the apple target was recognized and counted by the watershed segmentation and the circular Hough transform method (Bargoti and Underwood, 2017), where F1-score reached 85.8%. To accurately locate the tomato fruit under the complex scenes, Liu improved the YOLOv3 one-stage target detection model to predict the circular areas (Liu et al., 2020), and the precision and times of YOLO-Tomato were 94.75% and 54 ms. Jia optimized the Mask R-CNN to adapt to the detection of apple targets, where the residual neural network (ResNet) and dense convolutional network (DenseNet) were combined as the feature extraction network of the original model that the precision and recall rate have reached 97.31 and 95.70%, respectively (Jia et al., 2020b). Li proposed an ensemble U-Net segmentation model, and the high-level semantic features of U-Net and the edge features of Edge were integrated to retain multi-scale contextual information and realize efficient segmentation of target fruit (Li et al., 2021), where the recognition rate reached 95.11% and the recognition speed was 0.39 s. A modified YOLOv3 model based on clustering optimization is designed, and the influence of front-lighting and backlighting is clarified to detect and recognize banana fruits, inflorescence axes, and flower buds by Wu et al. (2021). To recognize and detect plant diseases, Chen et al. (2022) proposed an improved plant disease-recognition model based on the YOLOv5 network model *via* a new involution bottleneck module, an SE module, and an efficient intersection over union loss function to optimize the performance of target detection, where mean average precision reached 70%. A DaSNet-V2 network structure was proposed by Kang, visual sensors were used for real-time detection and instance segmentation of orchard apples, and the branches were segmented. Visual sensors were applied in field trials, and the experimental results showed that the presented method was efficient and reliable (Kang and Chen, 2020), where the precision of detection, fruit segmentation, and branch segmentation achieved 0.844, 0.858, and 0.795, and computational time was 30 and 265 ms on GTX-1080Ti and Jetson-TX2, respectively. To improve the performance of apple detection, a deep learning method approach based on the adaptive training sample selection (ATSS) was applied to close-range and low-cost terrestrial RGB images (Biffi et al., 2020). In addition, considering the lack of public datasets in the field of fruit detection, the MinneApple benchmark dataset (Häni et al., 2020b) and KFuji RGB-DS dataset (Gené-Mola et al., 2019) are publicly used to study fruit detection and segmentation. The MinneApple benchmark dataset is designed and published for detection and segmentation. The KFuji RGB-DS dataset is presented, containing 967 multi-modal images and 12,839 Fuji apples. Although the recognition accuracy of the target fruit is high based on deep learning, it has high requirements for machine hardware and requires a large number of training samples. In the actual operation process, it is difficult to meet these conditions one by one.

For the above problems, with the help of gradient information of depth images, an improved density peak cluster segmentation method of RGB images is proposed to fast location and accurate recognition of green apples based on RGB-D image information. The depth image of the apple is used to obtain the center of the target fruit and determine the location of the target fruit. To obtain the target fruit area, an optimized density clustering segmentation algorithm is introduced to segment the RGB image of the apple. Next, the depth and RGB images are combined by scanning the maximum radius of the segmented area where the circle center is located, and the target fruit contour is fitted to realize the efficient recognition and location of green apples. The new method applies the process of locating the circle center first and then recognizing, breaking the traditional method of recognizing first and then locating. Meanwhile, the new method is designed without iteration and a classifier, which effectively improves the efficiency of recognizing and locating the target fruit. In addition, the new method can complete the algorithm training for small sample datasets, greatly saving sample processing time. To summarize, our contributions to this article are as follows.

(1) The depth image information is applied to locate the fruit center *via* the idea of vorticity.
(2) An optimized density peak cluster segmentation method based on kernel estimation is designed to segment RGB images without subjective judgment.
(3) Experimental results outperform the other state-of-the-art models in accuracy and efficiency for green fruit recognition and detection.

The rest of this article is organized as follows. In section “Materials and Methods,” image acquisition and ideas of this article are introduced. In section “The Center Location of the Target Fruit,” we describe in detail how to use depth information to locate the target fruit. Section “Target Area Segmentation” illuminates how to apply color image information to segment the target. Section “Fitting of Target Fruit” is to fit the target fruit according to the fast-locating result of the depth image and the segmentation result of the color information. In section “Results and Discussion”, the experiments, including the experimental design and result analysis, are conducted and experimental results can be discussed. The conclusion is presented in section “Conclusion.”

## Materials and Methods

### Image Acquisition

Image acquisition location: Longwangshan apple production base in the Fushan District, Yantai City, Shandong Province (the agricultural information technology experimental base of Shandong Normal University). All images were collected under natural light.

Image acquisition device: Kinect V2 (Microsoft). The RGB images have a resolution of 1,920 by 1,080 pixels whereas the depth images have a resolution of 512 by 424 pixels. All RGB images and depth images were stored in the bitmap (BMP) format. The distance range from the camera lens to the target is 0.5–4.5 m. When the Kinect camera acquired the depth image, it collected each point in the field of view and forms a depth image representing the surrounding environment.

Apple category: Gala. Fruits were in an immature status before harvesting.

**FIGURE 1:**
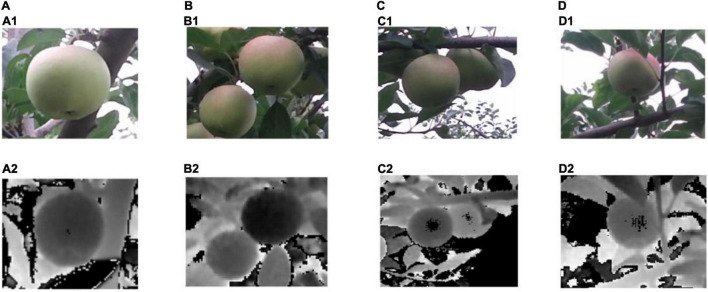
Apple images of RGB and depth images collected at the same sample point (note: panels **(A,B)** are single fruits without obscured; **(C)** are overlapping fruits; **(D)** are occlusion fruits; top images of every group represent the RGB image and bottom images represent depth images). The number of images: This study collected a total of 300 sample points data, including 300 depth images and 300 RGB images and containing 526 target fruits in the dataset.

Image acquisition mode: To imitate the actual monitoring environment, a tripod was used to fix the Kinect V2 camera angle. Kinect SDK 2.0 software was applied to capture the RGB-D images from the same angle. The color and depth images were saved in Portable Network Graphics (PNG) format. For the same fruits, we collected the images from different angles, including a single target, branch and leaf occlusion, or overlapped fruits. To facilitate image fusion, the resolution of all RGB images was adjusted to be the same as the resolution of the depth image 512 by 424 pixels. The images of green apples in actual environments, such as single fruits, overlapping fruits, and occlusion fruits, are shown in Figure 1.

### The Method and Ideas of This Article

**FIGURE 2:**
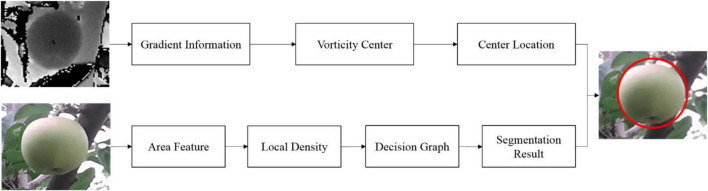
The overview of our method.

Based on the features of green apples, this article proposes a method for fast location and recognition of the green target fruit by the depth images (Choi et al., 2017) and RGB images of the target fruit. The depth image is utilized to find the center location of the target fruit and realize the effective location of the target fruits. Then, an RGB image is applied to segment fruits and background and obtain the target fruit area. Finally, the depth images and the RGB images are merged into a segmented area with the circle center as the target fruit, realizing the location and recognition of the target fruit. The overview of the new method is shown in Figure 2. The new method has two branches: locating the center of the target fruit through the depth image; segmenting the target fruit area from the RGB image.

For the depth image, the depth information of the target image is analyzed to draw an iso-contour map of the depth image and obtain the gradient field information of the target image. The three-dimensional gradient information of the target fruit is projected to the two-dimensional. To achieve the accurate location of the target fruit, the two-dimensional gradient vector is rotated 90° in the clockwise direction. It found that vorticities of the target fruit are relatively regular and orderly, whereas background regions present divergent and irregular states. According to the geometric meaning of the depth image, the center of each vorticity is viewed as the center of the target fruit.

A density peak clustering segmentation algorithm based on kernel density estimation is proposed in RGB images. First, a kernel density estimation method is applied to calculate the local density and difference in each data point in a non-parametric way. Note that a super-pixel segmentation algorithm is applied to segment images into blocks to convert images from pixel-level representation to area-level representation, which can reduce the number of data points and the amount of calculation. Then, a decision graph is constructed to separate the cluster centers and other data points. Next, a double sorting algorithm is adopted to automatically find the cluster centers, achieving the green target fruit segmentation.

Based on the center of the target fruit and the target fruit segmented area, the center of the target fruit is merged with the segmented area. Specifically, the segmented areas located in the circle center are considered as the green target fruits; the maximum value method is employed to obtain the radius of the target fruit. Finally, the contour of the target fruit is fitted to realize the location and recognition of the target fruit through the circle centers and its corresponding radius.

## The Center Location of the Target Fruit

Traditional target fruit location is mostly to segment the target fruit area first and then find the circle center through morphological methods. The location, detection, and segmentation results mentioned by these methods in the Introduction section are not accurate enough and the calculation amount is relatively large. In complex unstructured orchard environments, the accurate location of the target fruit is still an unsolved problem, and the locating of the green target fruit is even more challenging. This study introduces the depth information of the target fruits and applies the spatial information of the images to find the centers of the green apples.

### Contour Image Acquisition

The Kinect camera can directly obtain the distance information between the target and lens. Based on the imaging principle of the depth sensor, the pixel closer to the camera has a smaller distance value. The smaller distance value of the target corresponds to the smaller pixel value. The larger distance value of the surrounding background corresponds to the larger pixel value. Therefore, the pixels of the same value in the depth image are fitted to a closed curve. These curves can be projected onto a two-dimensional plane. Finally, the depth contour of the target image is obtained, that is, the depth of the pixels on the same depth contour is equal.

**FIGURE 3:**
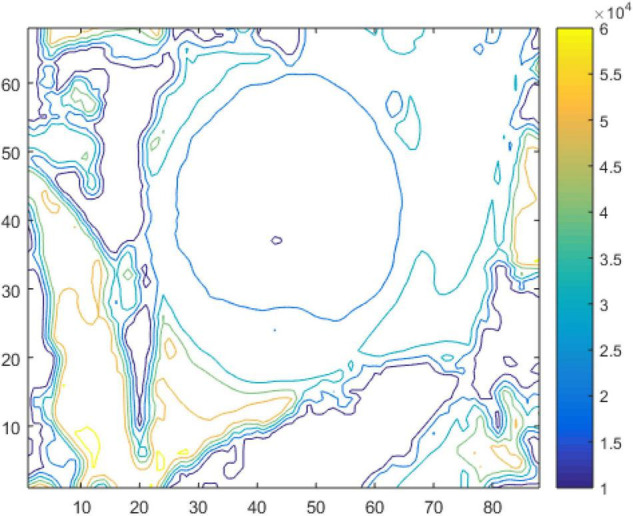
Contours of the depth image.

For the iso-contour map, a simple smoothing filtering process is performed. The three-dimensional geometric characteristics of the target are calculated through the mapped depth information. The depth of the image is drawn with a certain depth difference. Figure 1A will be an example to elaborate on the fast location and accurate recognition of green apples, as shown in Figure 3.

### Depth Image Gradient Information

**FIGURE 4:**
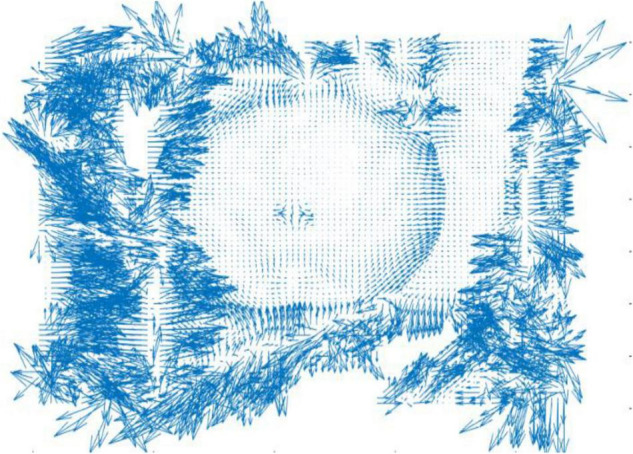
Two-dimensional plane projection of gradient field.

According to the principle of the depth sensor, the smallest pixel from the objects to the camera has the smallest distance value. As shown in Figure 3, the depth value of the central area of the target fruit is less than that of the non-central area of the target fruit based on the numerical characteristics of the depth image. Therefore, the depth information of the target depth image can be quantified by the gradient field theory. A vector $\vec{V}$ can be constructed.


(1) $$\vec{V}=\nabla D=\left(\frac{\partial D}{\partial x},\frac{\partial D}{\partial y},\frac{\partial D}{\partial z}\right)=\left(u,v,w\right)$$

where $\vec{V}$ represents a set of vectors (*u*,*v*,*w*) on the gradient vector field in three-dimensional space; *u*,*v*,*w* are partial derivatives of depth *D* in the *x*,*y*,*z* directions in three-dimensional coordinates, respectively, obtaining the gradient direction of the target fruits. Since the surface of the target fruit is convex, the 3D gradient field information can be projected onto the 2D plane. In other words, only the *x* and *y* directions are considered.


(2) $$\vec{V}=\nabla D=\left(\frac{\partial D}{\partial x},\frac{\partial D}{\partial y}\right)=\left(u,v\right)$$

where *u*,*v* represent the partial derivatives of the gradient field *D* in the *x*,*y* directions, respectively, which can obtain the gradient direction of the target fruit. Then, the direction vector on the 2D plane is obtained, which diverges outward along the apple surface in the gradient field, as shown in Figure 4.

In Figure 4, it can be found that the obtained direction vector presents a state of outward divergence in a relatively orderly way for the relatively regular target fruit area. Due to the complex background and the slightly disordered depth information, the vector projection is more chaotic in the non-target fruit area. In other words, the more regular the target fruit is, the more orderly the vector projection is. The more complex the background is, the more disorderly the vectors are.

### Center Location

Enlighted by dynamic vorticity, we apply the vorticity center of the target depth image as the fruit centers. Specifically, the vorticity is a 3D vector, and a pixel angular velocity (only direction) is introduced through the adjacent vectors.


(3) $$\vec{\omega^{\prime }}=\nabla \times \vec{V}=\left(\frac{\partial }{\partial x},\frac{\partial }{\partial y},\frac{\partial }{\partial z}\right)\times \left(u,v,0\right)=\left(\frac{\partial u}{\partial x}-\frac{\partial v}{\partial y}\right)\,\vec{z}$$

where ∇ is a partial derivative operator in *x*,*y*,*z* directions, and $\vec{z}$ represents a direction vector on the *z*-axis.

Since the rate of change of the vortex size increases from the edge to the center of the circle, the vectors in the gradient field are spliced into a similar arc shape. If the vector size is the same, the angular velocity is defined as follows:


(4) $$\omega =\frac{\phi }{t}$$

where ϕ represents the size of the arc length (similar to the size of the vector), and *t* represents the same time.

**FIGURE 5:**
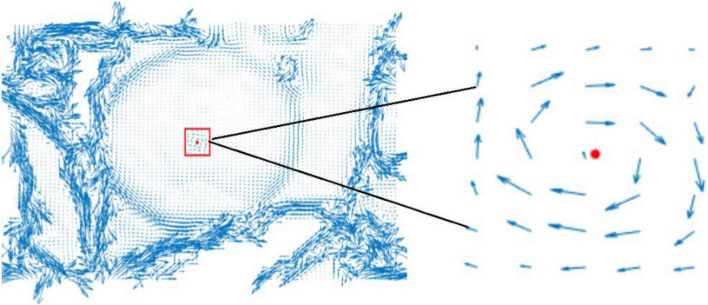
Location of target fruits circle center (gradient vectors are uniformly rotated 90° clockwise).

When gradient vectors with convex parabola are rotated in the same direction, a similar convergent vortex is formed in the position of vorticity maximum value and fruit center. In other words, the gradient vector of the primitive concave paraboloid is rotated 90° in a clockwise direction, and it becomes divergent and disordered. The maximum vorticity of the fruit area can be calculated at the center point of the fastest distance change, namely, the center of the target fruit. Further, the gradient vector of the fruit center area is a convex parabola center with such a characteristic: the vorticity of the central region of the fruit is greater than that of the surrounding region. In other words, the vorticity value of the fruit gradually decreases from the fruit center to the fruits boundary. Therefore, this geometric characteristic causes the gradient vector field plot of apples in 2D appeared to stretch out from the center of the apples to the direction of its closest perimeter pixel. The center of the vorticity can be projected onto a 2D plane, and the gradient vector is rotated in the same direction along the depth contour. The central location is the center of the target fruit, as shown in Figure 5.

It can be found that the vorticity centers of the regular area are the centers of the target fruits after rotation of the gradient vector in Figure 5. This method can rapidly locate the centers of the target fruits.

## Target Area Segmentation

For green apple image segmentation, it is difficult to achieve accurate segmentation by traditional image segmentation algorithms that only rely on color features due to the closeness of color between fruit skin and branches or leaves in the background. In this article, a density peak clustering algorithm is introduced to segment fruit area and background. To obtain a clearer segmentation boundary without human interference, a kernel density estimation method is utilized to optimize the clustering segmentation algorithm.

The main idea of the density peaks clustering algorithm (DPCA) (Rodriguez and Laio, 2014) is to judge the class that it belongs to by the neighboring points. The density of each class center point is greater than the density of neighboring points and this class center point is far enough away from the center points of other classes. Therefore, two main parameters are involved in the DPCA: the local density of data points and the distance between high-density points.

### Image Block Segmentation

The basis of the clustering algorithm is to calculate the distance between data points, which can be transformed into the description of the feature components of the data points, and the feature components determine the distribution relationship of the data points in the feature space. In the field of image segmentation, the pixel points of the image are the data points in the clustering algorithm. However, pixels considered as the calculation unit consume a lot of calculation space and time. Therefore, an image is divided into blocks, and the image blocks are applied as data points for clustering segmentation, which can greatly reduce the amount of calculation. The most common method of image block division is super-pixel segmentation, which uses the simple linear iterative clustering (SLIC) (Achanta et al., 2012) super-pixel segmentation algorithm for gathering similar pixels in a small area to form irregular blocks.

Considering the small color difference between the target fruit and the background, we convert RGB color space into the L*a*b color space emphasizing color change before using the SLIC algorithm. The cluster center is initialized to *C_k_*, and the iteration step is initialized to *S*. *S* can be formulated as follows:


(5) $$S=\sqrt{\frac{N}{k}}$$

where *N* is the number of image pixels and *k* is the number of blocks. To prevent the cluster center from falling on the edge of the image, the gradient value of each pixel is calculated in the 3 by 3 neighborhood of the cluster center, and the center is moved to the pixel with the smallest gradient. Next, iterative optimization is performed. To save computing time, the color and spatial distance are calculated, where the distance *Dis* between each pixel and the cluster center is calculated the *2S* by *2S* the neighborhood around the center point.


(6) $$\left\{ \begin{array}{c} d_{c}=\sqrt{{\left(l_{i}-l_{j}\right)}^{2}+{\left(a_{i}-a_{j}\right)}^{2}+{\left(b_{i}-b_{j}\right)}^{2}}\\ d_{s}=\sqrt{{\left(x_{i}-x_{j}\right)}^{2}+{\left(y_{i}-y_{j}\right)}^{2}} \end{array}\right.$$

The distance *Dis* between each pixel and cluster center is constructed as follows:


(7) $$D\,i\,s=\sqrt{{\left(d_{c}\right)}^{2}+{\left(\frac{d_{s}}{S}\right)}^{2}\,m^{2}}$$

where *l*,*a*,*b* are Lab space values, *x*,*y* are pixel coordinates, *andm* is the maximum possible value of Lab space distance. Each pixel updates the image block to which it belongs. The cluster center is updated by averaging the pixels of the same block until the center point no longer moves.

**FIGURE 6:**
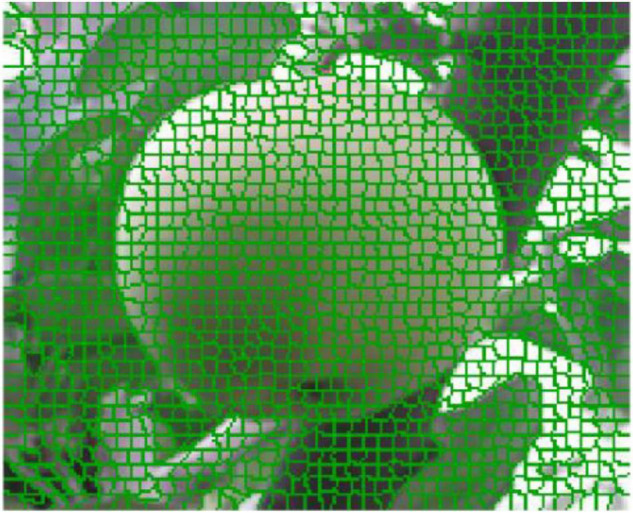
SLIC super-pixel segmentation effect.

The SLIC image block segmentation algorithm considers the color and space features of the image and defines the search range of *2S* by *2S* effectively reducing the computation complexity of the DPCA. Finally, the target image block effect map is presented, as shown in Figure 6.

It can be seen from Figure 6 that the image block is applied to represent the green apple image, which maintains the consistency of the original pixel points. These irregular blocks do not destroy the original image representation structure. Meanwhile, the target image is represented from the pixel level to the block level, which indicates that the conversion loss is small. Each irregular image block replaces pixels with the basic unit of the DPCA, which will greatly reduce the number of data points.

### Feature Component Selection

**FIGURE 7:**
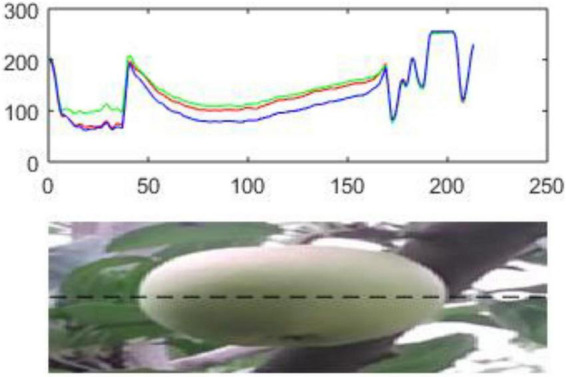
The RGB components corresponding to different scenes in the image (note: the red line represents the R component; the green line represents the G component; the blue line is the B component; the three lines are the RGB components corresponding to the pixels in the black horizontal dashed line).

After the target image is divided into image blocks, the effect of its shape and texture features is greatly reduced. Therefore, the feature component of the block is constructed in the unit of the super-pixel feature block as the feature components of the data points. Considering the closeness of color between the target fruit skin and the background, the color information of the target image is decomposed in the RGB color space to analyze features from different color channels to better select proper feature components, as shown in Figure 7.

**FIGURE 8:**
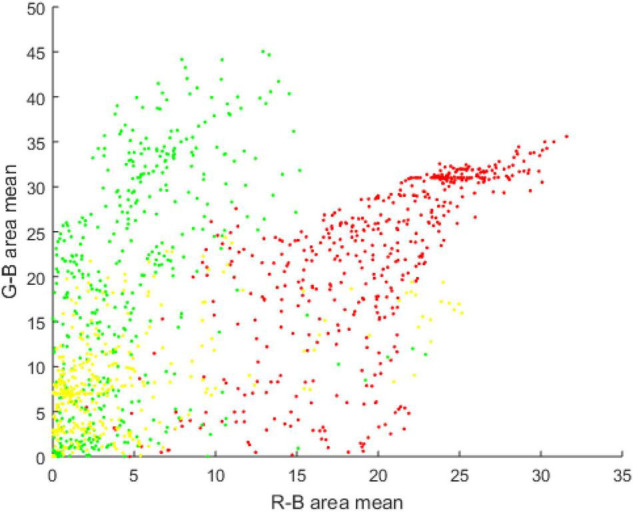
The feature space of the mean value of R-B and G-B.

It can be observed that the target fruit area in the green apple image has significant performance in the R and G channels, but is not obvious in the B channel in Figure 7. For the G channel, it is difficult to distinguish branches and leaves from the background. The performance of the B channel is not obvious for background branches and leaves and the target apple loses its significance. Therefore, R-B and G-B super-pixels are used to represent the image block features of the target image segmentation. Based on the image block, the R-B and G-B mean values are applied as the feature components of the data points to construct the feature space of the green apple target image through the density clustering segmentation algorithm, as shown in Figure 8.

The different colors of the data points represent the three classes obtained after clustering in Figure 8, where red, green, and yellow points represent green fruits, leaves, and branches, respectively. The boundary between the different clusters is not obvious and the shape is irregular. The data points of the boundary area are relatively scattered. Traditional clustering algorithms are difficult to deal with clustering problems with fuzzy boundaries, and low-density area boundaries are only presented inside the feature space.

### Density Peak Clustering Segmentation Algorithm

The DPCA algorithm can find out the high-density areas segmented by low-density areas and obtain arbitrary-shaped clusters. The cluster centers are fixed, where the data points belong to the clusters with the closest distance and greater local density. DPCA meets two assumptions: the local density of the cluster center (density peak point) is greater than the density of surrounding data points; the distance between different cluster centers is relatively far. To find the class center that meets the two conditions at the same time, the local density ρ_*i*_ of each data point *x_i_* and the distance δ_*i*_ from each data point *x*_*i*_to the data point *x*_*j*_are calculated, where the local density of the data point *x_j_* is greater than that local density of data point *x_i_* and the distance is the closest.

The distance between two data points is calculated by Euclidean distance and is written as follows:


(8) $$d_{i\,j}=\sqrt{\sum_{d=1}^{D\,i\,m}{\left(x_{i}-x_{j}\right)}^{2}}$$

where *Dim* represents the spatial dimension.

The local density ρ_*i*_ of the data point *x_i_* is defined as follows:


(9) $$\rho_{i}=\sum_{j}\chi \,\left(d_{i\,j}-d_{c}\right)$$

where *d_c_* is the cutoff distance that needs to be set manually. $\;\chi (x)\;=\;\{\begin{array}{cc} 1 & x\leq 0\\ 0 & x\text{>}0 \end{array}$, indicates the number of data points with a distance less than *d_c_* from the *i*-th data point as the true density of the data points *x_i_*.

The distance δ_*i*_ from data point *x_i_* to the nearest data point *x_j_* whose local density is greater than *x_i_* is defined as


(10) $$\delta_{i}=\left\{ \begin{array}{cc} \min\left(d_{i\,j}\right), & \rho_{j}>\rho_{i}\\ \max\left(d_{i\,j}\right), & \rho_{j}\leq \rho_{i} \end{array}\right.$$

The value with the smallest distance *x_i_* in the data points with larger local density than data point is found. If the local density of the data point *x_i_* is the largest, the maximum distance between *x_j_* and *x_i_* is selected.

Combining the above parameters, the basic principles and steps of DPCA segmentation are described as follows:

Step 1: the Euclidean distance *d*_*ij*_ between any two data points are calculated;

Step 2: the cutoff distance *d_c_* is set, and the local density ρ_*i*_ of data point *x_i_* and distance δ_*i*_ are calculated;

Step 3: a clustering decision graph is drawn with the local density ρ_*i*_ as the horizontal axis and the distance δ_*i*_ as the vertical axis;

Step 4: the data points with high values of ρ_*i*_ and δ_*i*_ are marked as cluster centers for the decision graph and points with relatively small values ρ_*i*_ but relatively large values of δ_*i*_ as noise points;

Step 5: each remaining data point is assigned to its nearest neighbor and the class of the data point with greater density until all data points are allocated.

The algorithm idea of DPCA segmentation is much more intuitive. It can quickly find the density peak point and can efficiently complete the sample allocation and noise point recognition. The segmentation algorithm can quickly complete clustering without iteration and has high operating efficiency. However, the segmentation performance is restricted by the cutoff distance to a certain extent. The selection of cutoff distance mainly depends on the human experience. Therefore, the selection of the clustering center has certain subjective factors.

### Kernel Density Estimation Optimization

The local density ρ of data points represents the distribution of neighboring data points in the area with the cutoff distance as the radius in the feature space. The selection of the cutoff distance will directly affect the clustering results. For the green target fruit, if the cutoff distance is not selected properly, it will be difficult to solve the boundary problem of the target image, so a more robust calculation method is needed. This study introduces a kernel density estimation optimization method to evaluate the local density of data points. This estimation is a non-parametric method, which can fully use its data characteristics to calculate, avoiding the influence of human prior knowledge. In other words, this method can get rid of the dependence on the cutoff distance *d_c_*.

The local density of data points is defined by kernel density estimation:


(11) $$\rho_{i}\left(x.y\right)=\frac{1}{n\,h}\sum_{i=1}^{n}K_{h}\left(\frac{x-x_{i}}{h},\frac{y-y_{i}}{h}\right)$$

where *K_h_* is the scaling kernel function, *h* > 0 is a smooth parameter. A Gaussian kernel function is adopted to smooth the peak function. Therefore, the scaling kernel function is


(12) $$K\,\left(x,y\right)=\frac{1}{2\,\pi \,\sigma^{2}}\,\exp\left(-\frac{{\left(x-x_{i}\right)}^{2}+{\left(y-y_{i}\right)}^{2}}{2\,\sigma^{2}}\right)$$

where, *x*_*i*_,*y*_*i*_ are the mean values of the R-B area and G-B area of the *i*-th image block, respectively.

**FIGURE 9:**
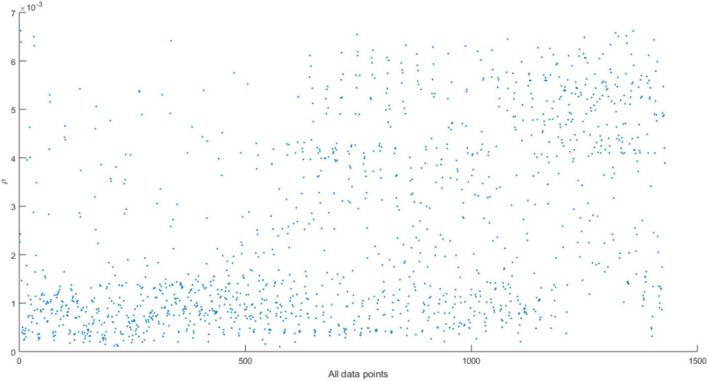
Local density distribution of target image data points.

The result of using Gaussian kernel density to estimate the density distribution of the green fruit target image is shown in Figure 9.

**FIGURE 10:**
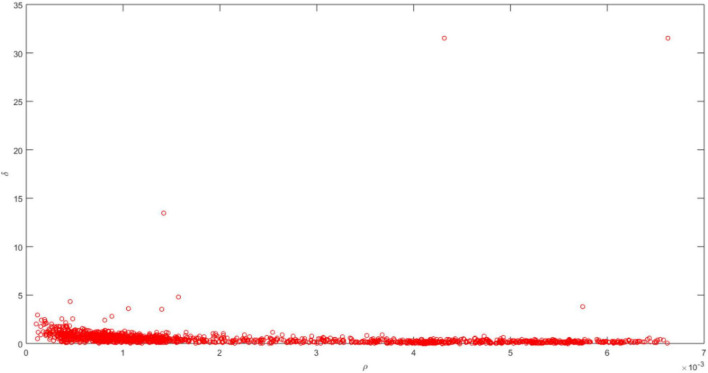
Decision graph of target image clustering.

Based on the local density ρ_*i*_ and distance δ_*i*_, the cluster center is a data point with a larger local density and distance. Thus, a decision graph with the local density ρ_*i*_ as the horizontal axis and the distance δ_*i*_ as the vertical axis is constructed for finding cluster centers, as shown in Figure 10.

It can be seen in Figure 10 that the data points with larger local density and distance in the decision graph are different cluster centers. However, the specific number of cluster centers needs to be artificially set according to actual problems. The data points close to the local density axis are cluster members and their distances are small. The data points close to the distant axis are abnormal points, and the data points in the upper right corner are outliers.

### Cluster Center Optimization

For the characteristics of the DPCA segmentation algorithm and the clustering process, the determination of non-cluster center data points does not completely depend on the cluster center, namely, the number of center points is not the number of final clusters. Considering that the complexity of the green apple image mainly lies in the complex structure between the target fruit and the background and the similarity of the two colors, it brings difficulties to the recognition of the target fruit. The segmentation of apple images is generally divided into three categories: target fruit, leaves, branches, and sky.

**FIGURE 11:**
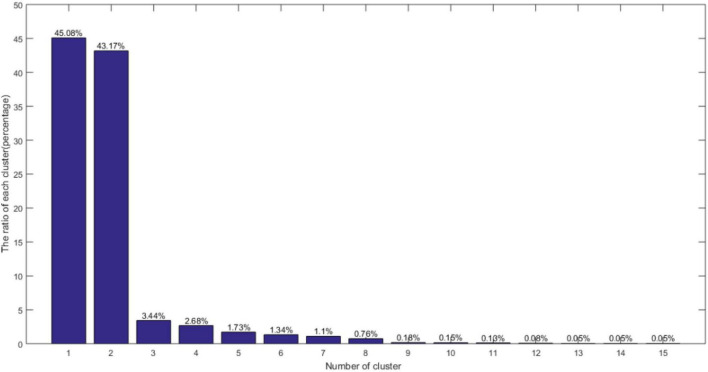
The ratio of the number of pixels in each class to the total number of pixels.

In this article, a double sorting algorithm is employed to automatically select the clustering center. Specifically, first, the local density value of all image blocks is sorted by descending and the first 20 image blocks are selected. Then, the distance values selected for 20 image blocks are sorted by descending order and the first 15 blocks are picked. Finally, the ratio of the number of pixels contained in each cluster and the number of pixels in the entire image is counted to analyze, as shown in Figure 11.

**FIGURE 12:**
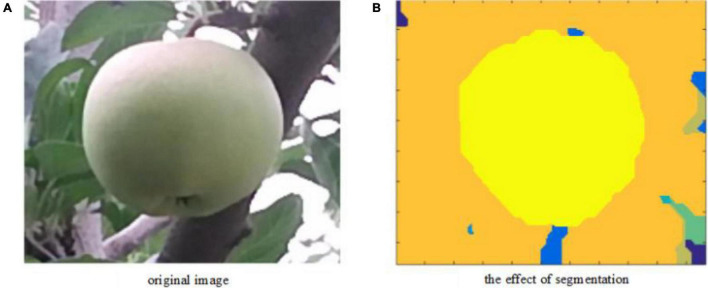
Target image segmentation effect graph. **(A)** The left image represents the original RGB image and **(B)** the right image refers to the visualization result of the density peak clustering segmentation algorithm based on kernel density estimation corresponding to the original RGB image.

It can be seen from Figure 11 that the first three categories account for 91.69% of the total number of pixels and the remaining categories of pixels account for a relatively low proportion. The clustering error of the target fruit area is brought by the boundary judgment and is related to the first 3 classes. The last 12 classes are regarded as the data non-allocation to the cluster center, which does not affect the target image segmentation. The segmentation effect obtained by the algorithm in this article is shown in Figure 12.

According to the segmentation result, the novel method completes the segmentation of the green target fruit and the background. The new method has a higher operating efficiency without iteration.

## Fitting of Target Fruit

In the apple image, the target fruit can be regarded as a round-like shape, which guarantees the two elements of the recognizing and locating of the round-like target: accurate locating of the circle center and value of the radius. Although green apples have brought new challenges owing to the close color of the target fruit’s skin and background, this study combines the depth and RGB image information of the green apple to solve the problem of recognizing and locating the green target fruit.

**FIGURE 13:**
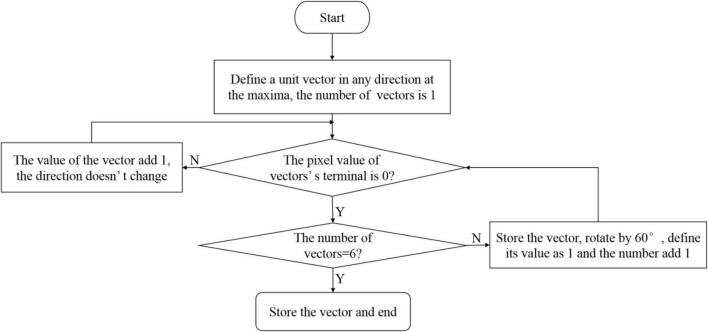
The process of determining the radius of the overlapping target fruit.

The center of the target fruit has been obtained in the depth image in section “The Center Location of the Target Fruit,” and the segmented area of the target fruit is obtained in the RGB image in section “Target Area Segmentation.” The above two results are merged. The target fruit area is the area where the center is located. In this study, the maximum value method is used to obtain the radius of the target fruit. Then, the contour of the target fruit is fitted to achieve the recognition and location of the target fruit. For a single target fruit, the maximum distance from the center of the circle to the edge of the region is directly selected. For overlapping target fruits, the minimum value from the extremum is used as the radius of the center. The process of determining the maximum radius of the overlapping target fruit is shown in Figure 13.

For overlapping target fruits, as shown in Figure 13, the segmented connected area containing the center of the circle is scanned to obtain six maximum vector lengths. The minimum value as the radius corresponding to the circle center is applied to realize the recognition and location.

**FIGURE 14:**
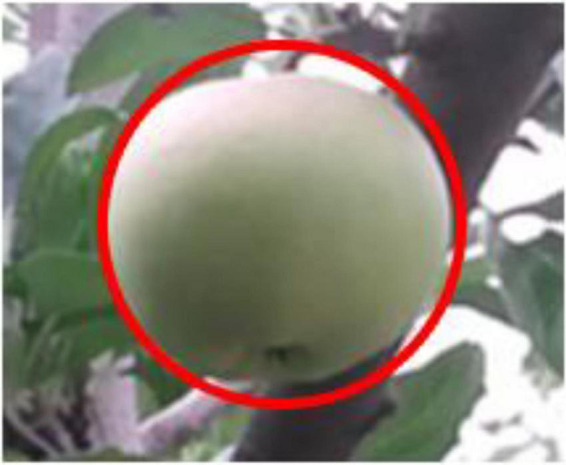
Fitting effect of target fruit.

Therefore, the center and radius of the circle have been determined, and the contours of the target fruit are fitted to complete the recognition and location of the target fruit, as shown in Figure 14.

After the new method completes the segmentation, there is no need to extract the features of the target fruit segmentation area, and there is no need to design a classifier. The target fruit can be determined by the central location. It can be concluded that the recognition accuracy and efficiency of the novel method are relatively high.

## Results and Discussion

Based on the above theoretical analysis, this research proposes a new method that can achieve rapid and accurate recognition and location of the target fruit of green apples. To better verify the effectiveness of the new algorithm, the following experiments are conducted.

### Result Assessment

The collected 300 pairs of green apple images were used in the experiment, including 526 target fruits. A single target fruit, occlusions of branches and leaves, and overlapping fruits are 287, 89, and 150, respectively. Recognition rate and running time are used to evaluate the recognition and location method. The recognition rates are calculated, including overall recognition rate, single target fruit recognition rate, obscured target fruit recognition rate, and overlapping target fruit recognition rate. The results of our optimized method with different growth postures are presented in Table 1. Note that the experimental operating platform of this study is as follows: the host configuration is equipped with an Intel Core i5-5257 CPU clocked at 2.7GHz and 4GB of memory; the operating environment is MATLAB R2015a installed with a 64-bit Windows 10 operating system. From Table 1, the total recognition rate of our method can reach 96.96%, where the recognition of fruits without obscurity is 99.3%. It can be concluded that our method has a high recognition rate.

**TABLE 1:** Correct recognition rate of three type’s fruits (%).

Model	Fruit without obscured	Obscured fruit	Overlapping fruit	Total recognition rate
**Ours**	**99.30**	**95.33**	**92.13**	**96.96**
Ref. 16	97.56	91.33	86.51	93.92
Ref. 12	97.91	89.33	85.39	93.35
Ref. 36	96.17	90.67	89.89	93.53

*Note that the bold value represents the optimal value under a specific evaluation metric.*

To better verify the performance of the new method, the experiment is compared with the method in the literature (Li et al., 2019; Lv et al., 2019a; Jiao et al., 2020). Meanwhile, the performance of the new method is evaluated from the above indicators. The results of comparative experiments with the literature (Li et al., 2019; Lv et al., 2019a; Jiao et al., 2020) are listed in Table 1.

Further, the operating efficiency and recognition accuracy of the whole new algorithm are evaluated, and the recognition time and recognition accuracy are calculated. The results are listed in Table 2.

**TABLE 2:** Comparison of the recognition performance of each algorithm.

Model	Recognition time (ms)	Total recognition rate (%)
**Ours**	**897**	**96.96**
Ref. 16	1269	93.92
Ref. 12	1505	93.35
Ref. 36	1627	93.53

*Note that the bold value represents the optimal value under a specific evaluation metric.*

It can be found from Tables 1, 2 that the efficiency of recognition and location of a single unobstructed fruit with a single branch and leaf obscured are the highest, and the recognition rate for overlapping target fruits is slightly lower. Based on the overall comparison effect, the recognition rate and operation efficiency have been greatly improved. The new method in this article is better than other methods.

### Visualization Result of Location and Recognition

**FIGURE 15:**
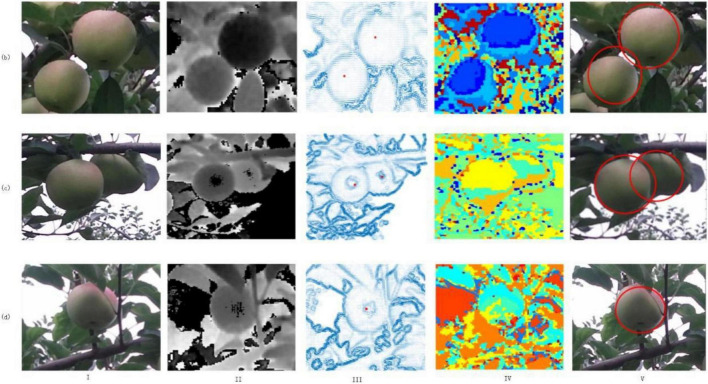
Three types of target fruit recognition and location effect (I) RGB space image, (II) depth image, (III) center position effect image, (IV) segmentation rendering, (V) identify and location.

Due to the variable natural growth posture of apple fruit, the shooting angle, and other factors, the collected target fruit presents three postures: single unobstructed, obscured by branches and leaves, and overlapping fruits. According to the new method in this article, three types of samples are tested, and the recognition results obtained are shown in Figure 15. We can find that a single unobstructed fruit, overlapping fruit, and fruit occluded by branches and leaves can all recognize and locate the target fruit.

### Result Analysis

It can be seen in Figure 15 and Tables 1, 2 that for the problem of green target fruit recognition and location, the new method has greatly improved the accuracy and operation efficiency of the recognition and location. The recognition rate of a single target fruit can reach almost 100%, indicating that the novel method is feasible.

In general, the novel method is designed without iteration during the clustering and segmentation process, without features of the segmentation area and a classifier during the recognition process. Therefore, the performance of the novel method is greatly improved. In terms of recognition and location accuracy, the new method can accurately locate the center and radius of the circle. The performance is also significantly improved.

### Limitation Discussion

**FIGURE 16:**
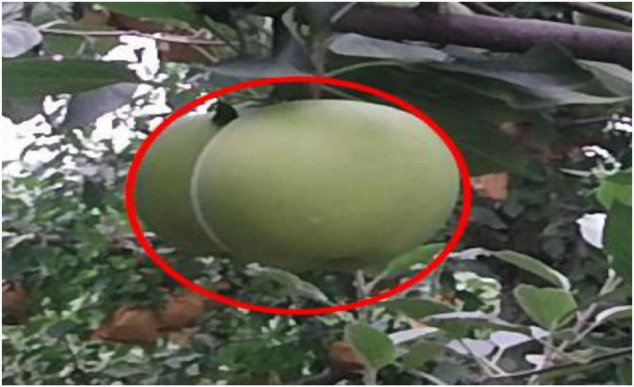
The visualization results of an excessively overlapping apple.

Although the overall recognition rate of the target fruit is relatively high, there is still a small problem: the recognition accuracy of excessively overlapping target fruits is low owing to the occlusion of branches and leaves for overlapping target fruits. Specifically, when the maximum value is used to find the radius corresponding to the circle center in the connected area of the segmented overlapping target fruit, the minimum value is scanned for the occluded area of branches and leaves, which makes it difficult to obtain the optimal radius. An excessively overlapping apple sample is shown in Figure 16.

For the problem of difficulty in locating the center of excessively overlapping fruit, we will consider optimizing the method of locating the center to reduce the dependence on depth information during the positioning process, that is, using a small amount of gradient information to achieve the method of locating the center of the circle. In addition, the RGB image segmentation method will be optimized to obtain smoother segmentation boundaries.

## Conclusion

Focusing on the problem of recognition and location of green apples, this article proposes a fast and accurate location and recognition method based on the fusion of depth images and RGB images. First, the gradient field information in the depth image is employed to draw an iso-level contour map and gradient vectors of the target image. All gradient vectors are rotated in the same direction to obtain different vorticity. The three-dimensional depth image is projected to the two-dimensional plane to find the center of vorticity. The center of vorticity is the center of the target fruit, achieving an accurate location of the center of the target fruit. Second, a density peak clustering algorithm is used to segment the target fruit in the RGB image. To get rid of the subjective factors of selecting a cutoff distance, the Gaussian kernel density estimation is applied to optimize the algorithm. For the cluster centers, a double sort method is utilized to select the cluster centers automatically to achieve efficient segmentation of target images. Finally, the results of the two steps are merged and the segmentation area of the circle center is just the target fruit area. The maximum value method is applied to obtain the target fruit radius, and the contour of the target fruit is fitted to complete the efficient and accurate recognition and location of the target fruit.

The proposed method is presented without iteration during the process of center location and clustering segmentation, without features of the segmentation area and a classifier in the recognition process. Therefore, the running efficiency of the new method is significantly improved. In the process of center location and radius calculation, the error is relatively small, and thus, the recognition and location accuracy of the new method is higher. Experimental results also show that the new method has greatly improved the accuracy and operating efficiency of recognition and location. In addition, our method is a lightweight method that does not require high-performance servers for computing, which can easily to transplant and embedded into the hardware environment of the fruit picking robot or applied to the automatic monitoring process of fruit growth. Therefore, the new method can solve the problem of recognizing and locating green target fruit well. The proposed method can be further extended to the problem of rapid and accurate recognition and location of spherical fruits, which can be used in the field of machine harvesting or yield estimation of spherical fruits. For the task of fruit recognition with overlapping targets occluded by branches and leaves, the radius calculation of the new method is susceptible to be interference, which will be the focus of future research.

## Data Availability Statement

The original contributions presented in the study are included in the article/supplementary material, further inquiries can be directed to the corresponding authors.

## Author Contributions

MS: conceptualization and writing—original draft preparation. LX: data curation, software, and validation. RL: funding acquisition and visualization. YL: methodology, software, and validation. WJ: conceptualization, funding acquisition, and writing—reviewing and editing. All authors contributed to the article and approved the submitted version.

## Conflict of Interest

The authors declare that the research was conducted in the absence of any commercial or financial relationships that could be construed as a potential conflict of interest.

## Publisher’s Note

All claims expressed in this article are solely those of the authors and do not necessarily represent those of their affiliated organizations, or those of the publisher, the editors and the reviewers. Any product that may be evaluated in this article, or claim that may be made by its manufacturer, is not guaranteed or endorsed by the publisher.
